# 3D microniches reveal the importance of cell size and shape

**DOI:** 10.1038/s41467-017-02163-2

**Published:** 2017-12-06

**Authors:** Min Bao, Jing Xie, Aigars Piruska, Wilhelm T. S. Huck

**Affiliations:** Institute for Molecules and Materials, Radboud University, Heyendaalseweg 135, 6525 AJ Nijmegen, The Netherlands

## Abstract

Geometrical cues have been shown to alter gene expression and differentiation on 2D substrates. However, little is known about how geometrical cues affect cell function in 3D. One major reason for this lack of understanding is rooted in the difficulties of controlling cell geometry in a complex 3D setting and for long periods of culture. Here, we present a robust method to control cell volume and shape of individual human mesenchymal stem cells (hMSCs) inside 3D microniches with a range of different geometries (e.g., cylinder, triangular prism, cubic, and cuboid). We find that the actin filaments, focal adhesions, nuclear shape, YAP/TAZ localization, cell contractility, nuclear accumulation of histone deacetylase 3, and lineage selection are all sensitive to cell volume. Our 3D microniches enable fundamental studies on the impact of biophysical cues on cell fate, and have potential applications in investigating how multicellular architectures organize within geometrically well-defined 3D spaces.

## Abstract (web summary)

Little is known about how geometric cues affect cell function and gene expression in 3D settings. Here the authors use microniches of different geometries to control cell volume and shape, and by extension cell phenotype and lineage.

## Introduction

Stem cells reside in vivo in a complex three-dimensional (3D) microenvironment, or niche, where multiple stimuli interact and integrate to regulate cell survival, self-renewal, and differentiation^1^. These stimuli include biochemical signals, such as growth factors and signaling molecules, as well as biophysical factors such as cell–cell and cell–matrix interactions^2^, matrix elasticity^3^, and geometry^4–7^. The integration of the various effectors is a complex but remarkably robust process, as evidenced, for example, by the fact that although different cell types can differ greatly in size and shape, within tissues cells are often strikingly similar^8^. Understanding how biophysical cues in the niche regulate stem cell function and fate is important, as it would lead to a much better insight into how cells develop and maintain their distinctive morphologies, and provide guidance for the design of new materials for tissue and organoid culture. Unfortunately, there are no in vivo methods to control niche geometry independent of changes in growth factors or other intra- and extracellular signaling events. Much of what we know about the influence of biophysical cues on stem cell fate comes from the cell culture studies on 2D micropatterned substrates^4–6,9–13^. These studies have provided a wealth of insight and have shown that cell geometry and size play an important role in organizing the cytoskeleton and in directing growth, death, and differentiation of mesenchymal stem cells (MSCs). However, 2D cell culture does not fully capture the cellular phenotypes found in vivo, cell volume cannot be controlled, and the inevitable polarization of cells spreading on adhesive substrates is a strong cue that cannot be decoupled from other parameters in the experiment. Surprisingly, culturing large numbers of individual stem cells fully enclosed in non-polarized and symmetrical 3D microniches with well-defined dimensions has not been achieved and how 3D size and geometry affects cell function remains elusive. To be sure, there has been important progress in capturing the physical aspects of the extracellular matrix by culturing cells within hydrogels^14–20^, but these gels present no geometrical restrictions on individual cells.

Here, we introduce a method to constrain stem cell size and geometry in a systematic and quantitative manner, by encapsulating cells in 3D hydrogel microniches: prism shapes with controlled geometries of the bottom plane and precisely defined volumes. This method allows for rapid acquisition of confocal microscopy images on large numbers of individual cells in identical microenvironment. We then present results on how size and geometry of 3D microniches affect actin polymerization, protein localization, gene expression, and lineage selection in human MSCs (hMSCs) with systematically increasing volumes and geometries with different aspect ratios (cubic and cuboid) and shapes (cylinder and triangular prism).

## Results

### 3D microniche preparation and single hMSC encapsulation

**Fig. 1:**
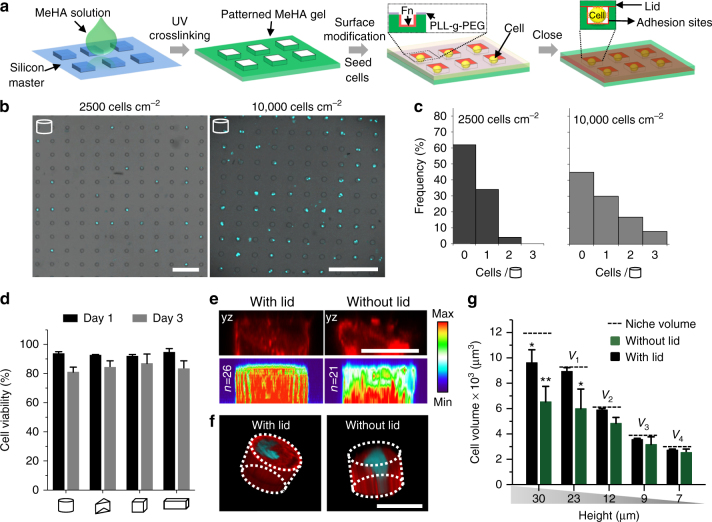
3D microniche preparation and single hMSC encapsulation. **a** Schematic of the method to encapsulate single cells in a 3D microniche. **b** Fluorescence image shows nuclear staining of single cells encapsulated in a 3D microniche with cylindrical geometries at different cell densities (2500 and 10,000 cells cm^−2^), scale bar: 100 µm. **c** Cell encapsulation efficiency at different cell densities in the 3D microenvironment with cylindrical geometry. **d** Quantification of cell viability (by live/dead staining) after 1 and 3 days of culture in a 3D microniche with different geometries; *n* ≥4 regions of interest (ROI) with a total of 80–100 cells analyzed. **e** Side view and fluorescent heat maps of actin organization in a microenvironment with and without lid, red: F-actin, scale bar: 20 µm. **f** 3D organization of actin cytoskeleton in a microenvironment with and without lid, red: F-actin, blue: nuclear, scale bar: 20 µm. **g** Quantification of cell volume after 24-h culture in a microenvironment with and without lid; *n* = 50–60 cells analyzed for each data point. The microniche volume was controlled by changing the height (from 7 to 30 µm), with a constant value for project area (400 µm^2^). Data are shown as mean ± s.d. for all panels, and *P < 0.05, **P < 0.01 (ANOVA using a Tukey post-test), compared to theoretical niche volume. Microniches with heights 23, 12, 9, and 7 µm are denoted as *V*_1_, *V*_2_, *V*_3_, and *V*_4_, respectively

The key to the successful design of 3D microniches is the requirement to fully encapsulate single cells within a matrix material that allows both cell adhesion and permeability of nutrients. Figure 1a shows our method for compartmentalizing cells in hydrogel niches with well-defined sizes and shapes. First, we formed wells in hydrogels of methacrylated hyaluronic acid (MeHA), a known biocompatible material (for synthesis and characterization see Supplementary Information and Supplementary Fig. 1), by photopolymerizing MeHA against a silicon master with patterns ranging between 5 and 40 microns in lateral dimensions and 7–35 microns in height. We can control the mechanical properties of these hydrogels between 1.8 and 36.5 kPa (Supplementary Fig. 2), although in this study we will focus on the impact of size and geometry of the microniches. Prior to seeding the cells, the hydrogel top surface was rendered protein-resistant using poly(L-lysine)-graft-poly(ethylene glycol) (PLL-g-PEG), deposited using a wet-stamping technique^12^. Subsequently, we soaked the PLL-g-PEG-modified wells with a fibronectin (Fn) solution (100 µg/mL), which binds directly to hyaluronic acid, to promote cell adhesion and spreading^21^. We achieved selective and uniform Fn deposition on the inside surface of the wells, as shown by confocal fluorescence microscopy after staining with a fluorescent antibody against Fn (Supplementary Fig. 3a, b). The Fn coating is also homogeneous within 3D microniches after closing the lid (Supplementary Figs. 3c, d). Supplementary Fig. 3e shows that over 96% of cylindrical microniches with different sizes were uniformly coated with Fn. hMSCs were deposited into the microniches by seeding on top of the patterned gel surface, followed by gentle shaking, and incubation at 37 °C for 10–15 min. Excess cells were removed by gentle washing with cell culture medium several times. To determine the efficiency of the seeding process, we counted cells by staining nuclei with DAPI (4,6-diamidino-2-phenylindole) (Fig. 1b and Supplementary Fig. 4a). When the cell density was too high (10,000 cells cm^−2^), cells were present on the surface of the MeHA hydrogel between microniches, requiring extensive washing with medium buffer, risking removal of cells from wells. Cell seeding density at 2500 cells cm^−2^ was optimal, with ~37% of wells filled with cells and over 95% containing single cells (Fig. 1c). Finally, and most importantly, to complete 3D encapsulation of the cells, we covered the wells with a flat piece (~30 µm thick) of MeHA hydrogel coated with Fn to construct a 3D microniche. Confocal imaging (Supplementary Fig. 4b) showed that the MeHA hydrogel lid fully sealed the wells. To confirm the permeability/diffusion of the cell culture medium through the MeHA hydrogel, we monitored the diffusion of green fluorescent protein (GFP) through the lid. After immersion in cell culture medium with GFP for 10 min, the fluorescence intensity of GFP protein inside the niches increased (Supplementary Fig. 4c), providing strong indication that key components of the cell culture medium could diffuse through the MeHA hydrogel. We measured the viability of hMSCs encapsulated in sealed 3D microniches via live/dead assay. After 24-h culture, over 90% cells remained alive in 3D microniches with different geometries (cubical, cylindrical, triangular prismatic, and cuboid) (Fig. 1d), and over 80% cells were still alive after 3 days of culture. After 10 days of culture, over 70% cells were still alive (Supplementary Fig. 5). These data confirmed that our 3D microniches maintain cell size and geometry for extended culture times. Compared with cells on 2D MeHA substrates, cell proliferation within 3D microniches was suppressed (Supplementary Figs. 6a and b). After 1-day culture, less than 5% microwells contain two cells (Supplementary Fig. 6c). We would like to stress the crucial differences between our 3D microniches and previous work on cells cultured in open microwells^16,22,23^ and 2D patterns^5, 9, 11, 13^. Fluorescence intensity heat maps on cells (*n* = 21 to *n* = 26) cultured in hydrogels with and without lid show that only in the fully enclosed 3D niches, the F-actin filaments were homogenously distributed from top to bottom (Fig. 1e and Supplementary Fig. 7). Three-dimensional reconstructions showed that cells completely filled 3D microniches with lids, but without lids, the surface of cells was irregular (Fig. 1f), and their volumes never matched the volume of the mold (Fig. 1g). This clearly shows that cells in wells without lids will not have uniform volumes, and will polarize due to the lack of integrin binding at the top surface (Supplementary Fig. 8); therefore, such substrates are not suitable for probing the impact of geometry of the 3D microniche on cell behavior. Control experiments also showed that without Fn coating, cells showed significantly less spreading and did not fully occupy the 3D niches (Supplementary Fig. 9).

### Stress fibers and F-actin polymerization in a 3D microniche

**Fig. 2:**
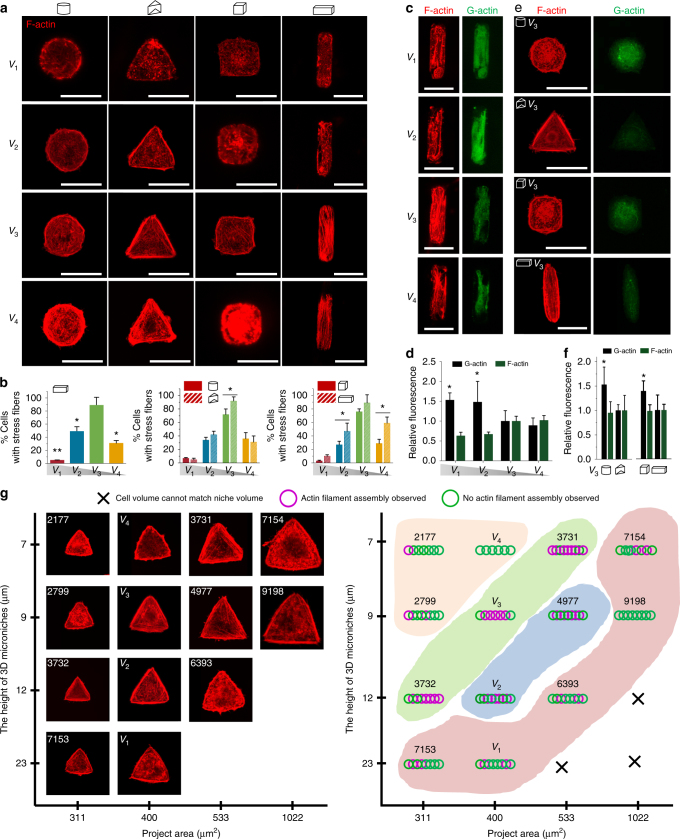
F-actin filaments formation and polymerization in a 3D microniche. **a** Representative images of F-actin staining for hMSCs with different cell volumes and cell geometries after 24 h. **b** Quantification of the number of cells forming stress fibers in a 3D microniche with different sizes and geometries; *n* = 50–60 cells analyzed for each data point. **c** Immunofluorescence images of F-actin and G-actin for hMSCs with different volumes after 12 h. **d** Quantification of F- and G-actin levels 12 h after seeding in 3D microniches with different volumes. Total integrated fluorescence of phalloidin (F-actin) and DNaseI (G-actin) was normalized to the fluorescence of *V*_3_ cells; *n* = 40–45 cells analyzed for each data point. **e** Immunofluorescence images of F-actin and G-actin for hMSCs with different geometries with *V*_3_ volume after 12 h. **f** Comparison of normalized mean F- and G-actin intensity in cells with different shapes (cylinder and triangular prism) and aspect ratios (cubic and cuboid); *n* = 40–45 cells analyzed for each data point. **g** Left: F-actin staining for single hMSCs cultured in 3D microniches with different volumes. Representative cells were selected for each condition. Right: quantification of the number of cells forming stress fibers in 3D microniches with different volumes. Colored regions show cell volumes between 2000 ~ 3000, 3000 ~ 4000, 4000 ~ 5000, and >5000 μm^3^, respectively. The values of cell volumes were presented on each image. Data are shown as mean ± s.d. for all panels, and *P < 0.05, **P < 0.01. Scale bar for all images is 20 μm

Experiments on 2D micropatterned islands have shown strong correlations between island geometry (shape, presence of sharp angles) and the organization of the actin cytoskeleton and focal adhesions (FAs)^13^. However, on such islands, cells are spread, volumes are not controlled, and the actin fibers are confined within a thin layer. We investigated the influence of cell volume on the organization of the cytoskeleton, by systematically varying the height of the 3D microniches (23, 12, 9, and 7 µm, respectively denoted as *V*_1_, *V*_2_, *V*_3_, and *V*_4_) while keeping the lateral dimensions fixed (400 µm^2^) (Fig. 2a). All these sizes were bigger than the average starting diameter and volume of hMSCs (~6.5 µm and ~2100 µm^3^, respectively) (Supplementary Fig. 10a and b), which means cells were able to spread and expand in the microniches and cell nuclei were not compressed initially in any of the microniches (Supplementary Fig. 10c). Surprisingly, as shown in Fig. 2b, in the largest volume cells (*V*_1_), F-actin staining showed few stress fibers and no apparent organization. With the volume of cells decreasing, increasingly clear and organized stress fibers were observed, and the number of cells that formed stress fibers increased significantly in 3D microniches with *V*_3_ volume. However, fewer stress fibers were observed in the smallest size cells (*V*_4_) (Fig. 2c).

In addition to cell volume, cell geometry also impacted on F-actin organization. Cells cultured in 3D microniches with the same volume (*V*_3_) but different shapes (triangular prism and cylinder) and aspect ratios (cuboid 1:4 and cube 1:1) showed markedly different F-actin organization, with more stress fibers observed in triangular prism and cuboid cells. It is interesting to note that F-actin organization became insensitive to the changes in cellular shape (triangular prism and cylinder) in cells with greater (*V*_1_) or smaller volumes (*V*_4_) (Fig. 2c).

We, together with the Watt group, previously showed that the ratio between F- and G-actin was an important determinant of cell fate in keratinocytes on micropatterned islands^9^. Quantification of phalloidin (F-actin) and DNaseI (G-actin) fluorescence revealed significantly higher levels of F-actin and lower levels of G-actin in cells with niche heights of 9 µm compared to cells in microniches with larger heights (*V*_3_ vs. *V*_1_ and *V*_2_; Fig. 2d, e). For a fixed cell volume (*V*_3_), the G- and F-actin levels were also dependent of cellular shape or aspect ratio. Cells with triangular prism and cuboid geometry displayed 55% and 42% lower signals for monomeric G-actin and comparable signals for F-actin compared with cells cultured in cylindrical and cubical microniches (Fig. 2f). Next, we seeded cells in microniches with different project areas (1022, 533, 400, and 311 µm^2^) and different geometries, but the same height (9 µm), where the project area of 400 µm^2^ corresponds to the original *V*_3_ microniches. We evaluated the cellular responses by investigating stress fiber formation (Supplementary Fig. 11). We found that when the project area (and corresponding volume) was too large (1022 and 533 µm^2^) or too small (311 µm^2^), F-actin staining showed few stress fibers, and little organization of the cytoskeleton—in contrast to the original *V*_3_ microniches with a 400 µm^2^ project area (Supplementary Fig. 11a and b). To further confirm that cell volume, and not aspect ratio, project area, or shortest axis, is the main factor in determining actin filaments formation in 3D, we mapped the stress fiber formation in cells seeded in microniches with different project areas (1022, 533, 400, and 311 µm^2^) and different heights (7, 9, 12, and 23 µm) (Fig. 2g). Remarkably, clear stress fibers were found in volume ranges around 3600–3700 µm^3^ (close to *V*_3_), up to approximately 4800 µm^3^ (with fewer cells showing clear stress fibers), irrespective of project area or height. When microniches were too large (6393, 7153, or 7154 µm^3^) or too small (2177 µm^3^), very few cells with clear stress fibers were found.

### Size and geometry affect FAs formation and cell tension

**Fig. 3:**
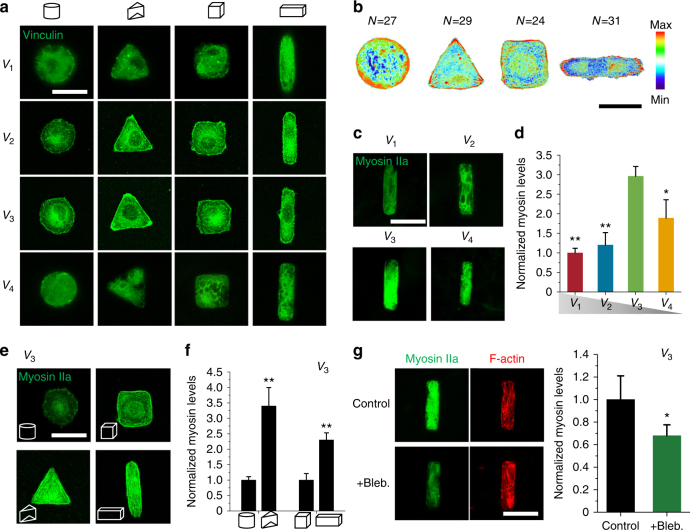
Focal adhesions formation and cell tension in a 3D microniche. **a** Representative images of vinculin staining for single hMSCs cultured in 3D microniches with different volumes and geometries. **b** Fluorescent heat maps of ≥20 cells with the same volume (*V*_3_) but different geometries stained for vinculin. **c** Representative images of myosin IIa in cells of same geometry but different volumes. **d** Myosin IIa levels (per cell) as a function of cell volume. **e** Representative images of cells with different geometries but same volume (*V*_3_). **f** Myosin IIa levels as a function of cell shape (cylinder and triangular prism) or aspect ratio (cubic and cuboid). **g** Representative images of myosin IIa and F-actin before and after cells with *V*_3_ treated with 50 μM Blebbistatin (Bleb); bar graph shows quantitation of the changes in the level of myosin IIa after treatment with 50 μM Blebbistatin (Bleb). Data are shown as mean ± s.d. for all panels; *n* = 45–60 cells analyzed for each data point and *P < 0.05, **P < 0.01 (ANOVA using a Tukey post-test). Scale bar for all images is 20 μm

We expect that the dependency of the formation of stress fibers on cell volume, and to a lesser extent geometry, will at least partially result from the localization of FAs. Unlike previous studies on 2D substrates, where more FAs were found in larger and spreading cells^24^, we only observed distinct FAs in *V*_3_ cells, and localization of vinculin (an FA-associated protein) to the periphery of the cells with different geometries was not observed in relatively large (*V*_1_) or small cells (*V*_4_) (Fig. 3a). To assess the differences in the patterns of FA between cells with different geometries, immunofluorescent heat maps were generated for >20 cells per geometry (Fig. 3b). Similar to findings on 2D substrates^13^, FAs were predominantly formed in regions of curvature in triangular prism cells or at the edge of cuboid cells with increasing aspect ratio. Immunofluorescent staining of myosin IIa, the primary motor protein assembly that is responsible for cell contractility and tension, was performed for hMSCs after 24-h culture. Cells with *V*_3_ and *V*_4_ were found to have higher levels of myosin IIa compared with *V*_1_ and *V*_2_ cells (Fig. 3c, d). For cells with a fixed volume (*V*_3_), myosin IIa intensity was strongly dependent on cell shape and aspect ratio (Fig. 3e, f). Perturbation of myosin IIa activity by 50 μM Blebbistatin (Bleb) resulted in a decreased formation of stress fibers (Fig. 3g), indicating that the enhanced stress fiber formation in niches with optimal size and geometry was regulated by cell contractility.

### Size and geometry affect nuclear function and TF activity

**Fig. 4:**
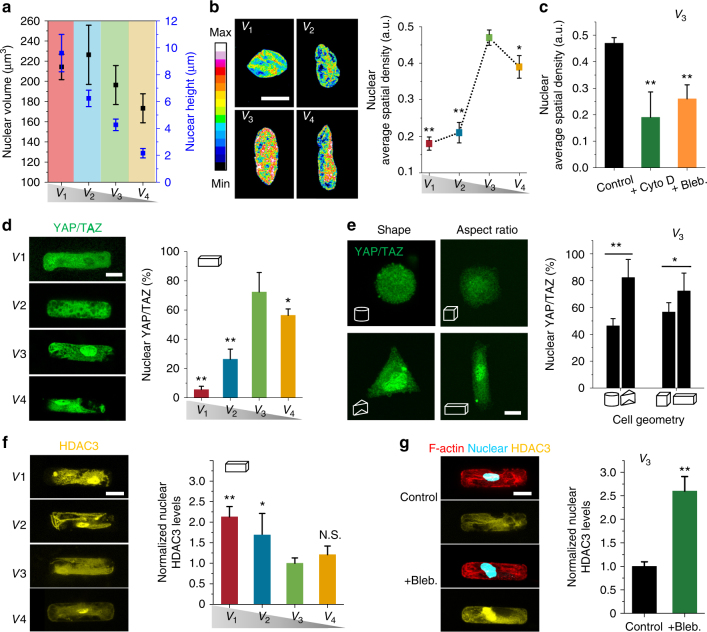
Nuclear function and transcription factor activity **a** Nucleus volume and height as a function of cell volume. The volume of nucleus was calculated by fitting the morphology of nucleus to an ellipsoidal shape. Data are given as mean ± s.d. with 12 ≤ *n* ≤ 15. **b** Quantitation of nucleus average spatial density (total DAPI intensity per nuclear volume) as a function of cell volume. Highly condensed domains show higher fluorescence intensity. The scale bar is 5 μm. **c** Quantitation of the changes in the level of nucleus average spatial density for cells with* V*_3_ after treatment with 1 μM cytochalasin D (Cyto D) or 50 μM Blebbistatin (Bleb). **d** Representative images and quantification of YAP/TAZ localization in hMSCs with different cell volumes but same geometry after 24 h. Scale bar 10 µm. **e** Representative images and quantification of YAP/TAZ localization in hMSCs with different cell geometries but same volume after 24 h. Scale bar 10 µm. **f** Representative images of hMSCs stained for HDAC3 on cells with different volumes but same geometry. Histogram shows nuclear HDAC3 levels as a function of cell volume. **g** Representative images and quantitation of cells with *V*_3_ treated with 50 μM Blebbistatin (Bleb). Data are shown as mean ± s.d. for all panels, *n* = 50–60 cells analyzed for each data point. *P < 0.05, **P < 0.01 (ANOVA using a Tukey post-test). NS no significant difference

Previous studies have shown that actin filaments play an important role in modulating nuclear shape and function^25^. We therefore expect to see nuclear deformation in cells with significant organization of actin filaments. Figure 4a shows the decreasing height of nuclei as a function of decreasing niche height from 23 to 7 μm. The volume of the nucleus was the largest (228 μm^3^) in cells of volume *V*_1_ and *V*_2_ (no significant difference), while the nucleus volume decreased significantly with decreasing cell size from *V*_2_ to *V*_4_. We examined the chromatin condensation by using a quantitative procedure based on DAPI staining^11, 25^. Figure 4b shows a marked reorganization of chromatin distribution associated with nuclear deformation, as the uptake of DAPI depends on the total amount of DNA, but also on its level of condensation. The average spatial density of nuclei first increased with decreasing niche heights from 23 to 9 μm, then decreased when the niche height reached 7 μm (Fig. 4b). To confirm the role of actomyosin filaments in the modulation of the nuclear architecture, we treated cells with cytochalasin D (Cyto D) or Bleb. As shown in Fig. 4f, both treatments significantly decreased the average spatial density of nuclei by ~50%, indicating that the actin filaments play an important role in modulating nuclear shape and function.

Next, we examined the nuclear localization of the YAP/TAZ transcriptional regulator, which is thought to be the key regulatory element controlling the gene expression of cells in response to physical cues^26^. Surprisingly, fluorescence staining (Fig. 4d) showed that YAP/TAZ remained cytosolic in cells in microniches with *V*_1_ and *V*_2_ volume, but was located in the nuclear region when the cell volume was *V*_3_ and *V*_4_. Nuclear translocation of YAP/TAZ increased from 52 to 70% with increasing cell volume from *V*_4_ to *V*_3_, but decreased to 5% with increasing cell volume to *V*_1_.

Next, we studied nuclear YAP/TAZ localization in cells in microniches of 9 µm height, but different volumes. Consistent with stress fiber results above, nuclear YAP/TAZ localization was strongly dependent on cell volume (Supplementary Fig. 12). In cells with *V*_3_, YAP/TAZ localization was also strongly dependent on cell geometry (shape and aspect ratio), cells with triangular prism and cuboid geometry showing 82% and 73% nuclear YAP/TAZ localization, compared with 46% and 57% nuclear YAP/TAZ localization in cells with cylinder and cubic geometry, respectively (Fig. 4e). Previous studies have shown that nuclear histone acetylation is regulated by actomyosin contractility and nuclear morphology^11^. We investigated the effect of cells in different 3D microniches on histone acetylation levels by analyzing histone deacetylase 3 (HDAC3)^27^, and found that nuclear levels of HDAC3 were lower in cells of *V*_3_ compared with larger (*V*_1_ and *V*_2_) ones (Fig. 4f). Perturbation of actomyosin contractility results in nuclear accumulation of HDAC3 (Fig. 4g), indicating that nuclear HDAC3 localization is sensitive to changes in actomyosin contractility.

### Size and geometry affect mRNA concentration in cells

**Fig. 5:**
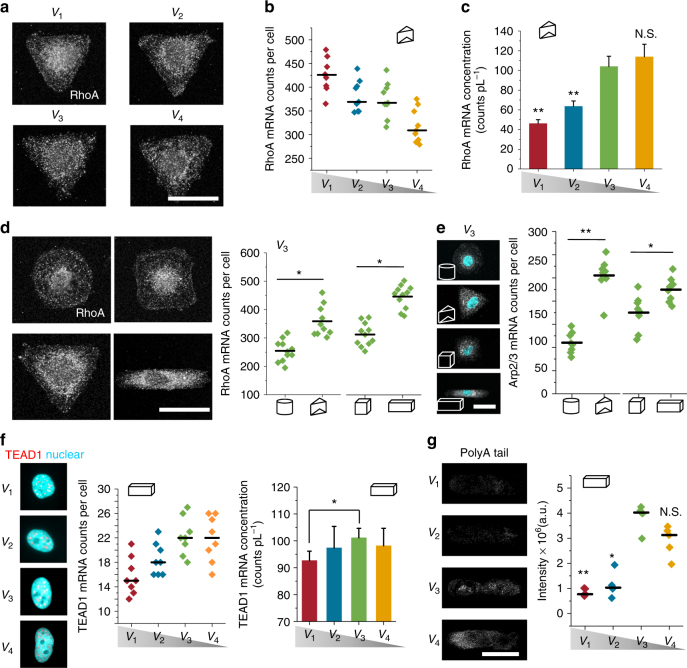
Size and geometry affect mRNA concentration in cells. **a** Representative images of RhoA mRNA in cells with different volumes. **b**, **c** Counts and concentration (divided by cell volume) of RhoA mRNA in cells with different volumes, narrow lines represent the mean within an individual donor; *n* = 10 cells per donor and condition. **d** Representative images and total counts of RhoA mRNA in cells with different geometries but same volume (*V*_3_). **e** Representative images and total counts of Arp2/3 mRNA in cells with different geometries but same volume (*V*_3_). **f** Representative images, counts, and concentration (divided by nuclear volume) of TEAD1 in cells with different volumes. **g** Total mRNA in the middle stack of cells with different volumes; we measured total mRNA by quantifying total fluorescence intensity from an mRNA FISH probe that detects polyA tail. Data are shown as mean ± s.d. for all panels; *n* = 30–35 cells analyzed for each data point. *P < 0.05, **P < 0.01 (ANOVA using a Tukey post-test). NS no significant difference. Scale bar for all images is 20 µm

Biochemical reaction rates depend on the concentration of reactants and enzymes^28^. To maintain proper cellular function, concentrations must be buffered against fluctuations in volume. The experiments above have shown that we can change the confinement in one dimension by changing the height of the microniches, but we can also compare cells with similar aspect ratios yet different volumes. To study the impact of changes in cellular concentrations of key components in cells with different volumes, we quantified gene expression levels of specific genes using single-molecule multicolor mRNA fluorescence in situ hybridization (RNA FISH). Specifically, we monitored the expression of RhoA, Arp2/3, and TEAD1. RhoA is a protein that can stimulate formation of actin stress fibers, FAs, and cytoskeletal tension, and shape-dependent control of lineage commitment is mediated by RhoA activity^4, 29^. We found that copy numbers of RhoA mRNA increased with increasing cell volume from *V*_4_ to *V*_1_ (Fig. 5a, b), and also depend on cell geometry (Fig. 5d), a similar effect was found on 2D substrates^4^. However, the concentration of RhoA mRNA was significantly lower in larger cells (Fig. 5c), indicating that RhoA was diluted in large cells. To validate this, we stained RhoA-GTPase with active RhoA-GTP monoclonal antibody. Consistent with the mRNA result, we found that total RhoA-GTPase intensity decreased (slightly, by a maximum of 20%) with decreasing cell volume from *V*_1_ to *V*_4_. However, the intensity of RhoA per stack was the largest for cell volume *V*_3_ (Supplementary Fig. 13a and b), indicating that RhoA was diluted in larger cells. To explore the function of Rho in regulating cell behavior, C3-exoenzyme was used as a specific inhibitor of Rho GTPase; inhibition of Rho activity resulted in decreased formation of stress fibers (Supplementary Fig. 13c). The diluted RhoA in larger cells might thus impact cell behavior. The actin-related protein-2/3 (Arp2/3) complex is a central protein in regulating actin filament formation^30^, and the activity of Arp2/3 has been shown to strongly depend on RhoA^31^. Consistent with the results for RhoA, we find that cells with prism and cuboid shape have higher Arp2/3 mRNA copy numbers (Fig. 5e). Thirdly, we studied mRNA levels of TEAD1, a nuclear transcription factor that forms ternary complexes with YAP/TAZ^32^. We found highest TEAD1 mRNA copy numbers in smaller cells, while the concentration of TEAD1 mRNA in *V*_3_ cells reach the highest level (Fig. 5f). Previous studies have shown that mRNA concentration is typically higher in smaller cells, sometimes by a factor of two or more^28^. By detecting polyA tails, we found that the total mRNA intensity (which in this experiment equals concentration) in *V*_3_ cells was four times higher than cells with *V*_1_ cells (Fig. 5g).

### Size and geometry affect single hMSC fate

**Fig. 6:**
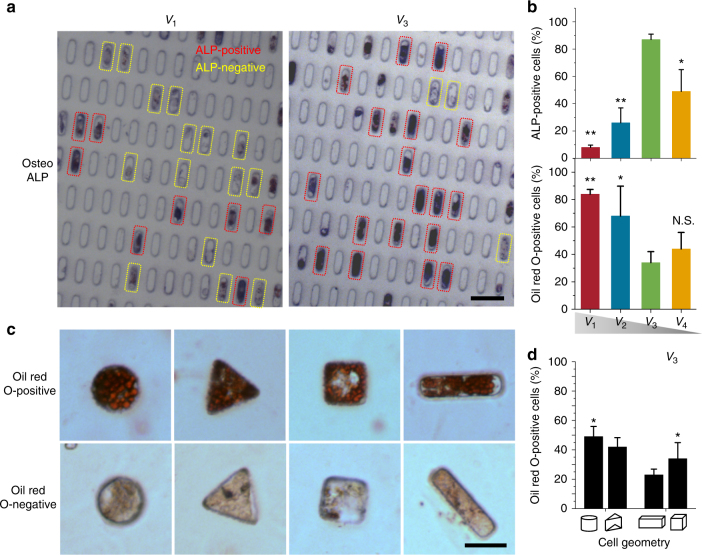
Size and geometry affect single hMSC fate. **a** Alkaline phosphatase (ALP) staining for cells with different *V*_1_ and *V*_3_ volume. The ALP-positive cells were determined by applying an optimal threshold to the image; ALP intensity above the threshold was determined as ALP positive. **b** Quantification of differentiation after 7 days (ALP) and 10 days (Oil Red O) for cells with different volumes. **c** Representative images show Oil Red O positive and negative staining for cells with volume (*V*_3_) but different geometries. **d** Quantification of adipogenic differentiation after 10 days for cells with different geometries. Mean ± s.d., ANOVA one-way analysis followed by Tukey post-hoc test shows significance levels of *P < 0.05, **P < 0.01. NS no significant difference. *N* ≥ 6 regions of interest (ROI) with a total of 150–200 cells analyzed. Scale bar 20 µm

Finally, we studied differentiation of hMSCs in 3D microniches; 82% and 67% of *V*_1_ and *V*_2_ cells, respectively, exhibited adipogenic differentiation, as indicated by staining for neutral lipids, and very low levels (less than 26%) of osteogenic differentiation, as indicated by alkaline phosphatase staining (Fig. 6a, b and Supplementary Fig. 14). Osteogenic differentiation was significantly enhanced (87%) in cells of *V*_3_; however, when cell size decreased to *V*_4_, decreased osteogenesis and increased adipogenesis were observed, and compared with *V*_1_ and *V*_2_ cells, adipogenic differentiation in *V*_3_ cells was significantly decreased (Fig. 6b). For hMSCs with fixed cell volume (*V*_3_), but different geometries, we found that cylinder and cubic geometry induced more differentiation into adipocytes, compared with cells in triangular prism and cuboid microniches (Fig. 6c). In cylinder and cubic cells, approximately 40–60% and 30–50%, respectively, of cells stained positive for neutral lipids (Fig. 6d). These experiments illustrate that 3D geometry and size play a very important role in regulating cell fate.

## Discussion

The 3D microniches we have described here allow us to control both cell volume and geometry. They differ from previous studies on 2D micropatterned substrates and microwells, as they fully enclose cells, providing a completely non-polarized microenvironment of precisely defined volume. A key observation is the remarkable impact of cell volume on F-actin self-organization. Although our 3D microniches are still grossly oversimplified compared with the in vivo scenario, our results on 3D microniches with different aspect ratios, but similar volumes, clearly show the decisive influence of cell volume on stress fiber and FA formation, within the range of sizes studied. Our results on Rho-GTP and mRNA levels within cells of different volumes indicate that although cells may have similar amounts of protein or mRNA, their concentration might be different. Volume changes, possibly as a result of the influx of water^33^, can thus lead to differences in interactions between key regulatory proteins in the cell. Previous studies have shown that mRNA concentration is typically higher in smaller cells, sometimes by a factor of two or more^28^; however, the consequences of this effect, in particular on cell function, are not very clear. We found that RhoA, Arp2/3, TEAD, and total mRNA concentration were diluted in large cells. It is interesting to note that this dilution of RhoA and Arp2/3, both playing a role in the formation of actin fibers, correlates with a much less pronounced organization of the actin cytoskeleton and higher proportion of G-actin in larger cells (Fig. 2e). The observed increase in actin organization in some of the higher aspect ratio structures might be related to the fact that actin forms relatively stiff fibers with persistent lengths in the range of 5–10 µm^34–37^. Recent experiments suggest that the organization in semiflexible polymer networks, including those consisting of actin and actomyosin, indeed responds to the geometry of the confining containers^38–40^.

Our experiments also suggest that much of the further cellular response follows cytoskeleton organization. We demonstrated a strong coupling between cell geometry, actin polymerization, FA formation, and actomyosin contractility. Immunofluorescence analysis showed higher levels of organized FAs and cell tension in cells with volume *V*_3_ and anisotropic geometries such as triangular prism and cuboid cells. Maintenance of nuclear architecture is essential for many cellular processes like transcription and protein synthesis^11,25^. By varying the volume of the cells, we observed changes in morphological properties of the nucleus volume. This suggests that geometric cues are translated into long-range physical forces that alter nuclear morphology. We also found that perturbation of actomyosin contractility resulted in nuclear accumulation of HDAC3, consistent with literature reports^11^. It should be noted that previous literature reported that HDAC3 activity will give rise to chromatin condensation^11^; the apparent correlation between HDAC3 levels and chromatic condensation in this study might indicate that other factors are altering chromatin condensation against the observed trend for HDAC3. Furthermore, changes in volume and geometry directly impinge on the distribution of YAP/TAZ: we observed maximum nuclear localization of YAP/TAZ in cells with *V*_3_ volume, and in cells with more anisotropic or sharp-angle-containing geometries, correlating with the fact that in those cells the presence of stress fibers, FAs localization, and cell tension were all highest.

The control over cell volume, and the option to alter the mechanical properties of the gels, as indicated in the supporting information, enables further studies on the role of biophysical cues. Here, we have focused on single cells, but also at the tissue-level, geometry and biophysical factors play a significant role^41–43^. It is straightforward to investigate a range of other geometries, including asymmetric ones, and it will be very interesting to compare the response to geometrical cues between different cell types. We also foresee further broadening of the use of our platform by studying cell division^44^ and exploiting the ability to culture multiple cells, which makes it possible to explore how multicellular architectures such as tumor spheroids, organoids, or microtissues organize within geometrically well-defined 3D spaces.

## Methods

### MeHA synthesis

MeHA was synthesized following a previously described procedure^45, 46^. Briefly, methacrylic anhydride (MA) (Sigma) was added to a 1% w/v solution of sodium hyaluronate (HA, Lifecore, 70 kD) (2.4 mL MA per gram of HA) at pH 8.0 on ice for 8 h, and subsequently reacted with MA (1.2 mL MA per gram of HA) at pH 8.0 on ice for 4 h. The pH was adjusted with 5 M NaOH. The reaction mixture was dialyzed in deionized water (Spectrapor, molecular weight cutoff 3.5 kDa) at 4 °C for 3 days and lyophilized. ^1^H NMR (Bruker Avance III 400 MHz) was used to confirm methacrylation of hydroxyl groups on HA (Supplementary Fig. 1).

### Fabrication of MeHA microwells and lid

Microwells with different geometries and different dimensions were produced on a silicon mask using standard photolithography and inductively coupled plasma etching (ICP). The silicon master was silanized with 1H,1H,2H,2H-Perfluordecyltriethoxysilan (Sigma). The photoinitiator for the hydrogel, lithium phenyl-2,4,6-trimethylbenzoylphosphinate (LAP), was synthesized following a previously described procedure^47^. MeHA macromer and LAP were separately dissolved in PBS (pH 7.4) and mixed at room temperature to a final weight percentage of 9:1 respectively. Crosslinking of the gel was initiated by irradiation with a UV lamp (ABM, USA) at 25 mW cm^−2^ for 5 min with a light output of 30%. The gel was peeled off the silicon master and washed two times for 30 min in PBS to remove LAP. A lid was produced in a 24-microwell plate. A thin layer of the MeHA pre-solution (200 µL) with photoinitiator described above was crosslinked with the UV lamp for 5 min at 30% intensity. To visualize the MeHA lid cover on the microwells, 0.1 wt% rhodamine (Sigma) was added to the pre-solution to form MeHA hydrogel lid.

### Fabrication of polyacrylamide (PAAm) gel containing PLL-g-PEG

PAAm gel containing PLL-g-PEG was made on a glass coverslip (13 mm, thickness no 1, borosilicate glass); in order to make PAAm gel attach on the glass coverslip, coverslips were oxidized using oxygen plasma (Diener electronic) and then incubated in a 0.3 wt/vol% solution of 3-(trimethoxysilyl)propyl methacrylate (Sigma Aldrich) in dry toluene overnight. The slides were washed thoroughly with ethanol and water. Solutions of acrylamide (AA) at final concentrations of 30 wt/vol% and bis-acrylamide (BA) at 0.5 wt/vol% were prepared. Thirty-microliters of 1 mg mL^−1^ PLL-g-PEG (Sigma-Aldrich) in PBS was added to 1 mL PAAm pre-gel; polymerization was initiated by the addition of 10 µL of 10 wt/vol% ammonium persulfate (Sigma Aldrich) and 3 µL TEMED (Sigma Aldrich) to the AA/BA solutions in PBS. Five-microliters of the gel precursor solution was immediately pipetted onto de-methacrylated glass coverslips and a 20-mm glass coverslip, washed but untreated, was carefully placed on top of the polymerizing solution. After 2 h, the samples were soaked in PBS overnight to remove the remaining monomer and crosslinker. The top coverslips were peeled off to obtain the PAAm gels containing PLL-g-PEG adhering to the glass coverslips.

### MeHA hydrogel functionalization with fibronectin

The space in between the microwells on the MeHA hydrogel was made resistant to protein adsorption and cell adhesion with PLL-g-PEG. The PLL-g-PEG was attached to the hydrogel following a microcontact printing procedure. The MeHA hydrogel containing the microwells was placed upside down on the PAAm gel (fully dried) containing PLL-g-PEG for 1 h with a 10-g weight. To increase the amount of transferred PLL-g-PEG, the procedure was repeated up to six times. Afterward, Fn (Sigma-Aldrich) solution at 100 µg mL^−1^ was added on the surface of the MeHA gel for 1 h at room temperature, followed by washing three times with PBS to remove extra Fn solution. Fn inside microwells was stained with anti-Fn antibody (Abcam, ab2413, 1:1000). For MeHA hydrogel lid preparation, same protocol was used but without PLL-g-PEG treatment.

### Hydrogel mechanical characterization

In order to show the possibility of changing stiffness for MeHA hydrogel, different MeHA concentration was used to obtain gels with a range of crosslinking densities. The stiffness of gels was measured by nanoindentation under an atomic force microscope (Bruker Nanoscope) using the “point and shoot” procedure (Nanoscope software, Bruker) as we reported previously^48^. A fluorescent polystyrene bead with a 10-μm diameter (Invitrogen) was glued to silicon nitride cantilevers with a nominal spring constant of 0.06 N m^−1^ (NP-S type D, Bruker). We calibrated the system in a cell-free medium at 37 °C prior to each experiment by measuring the deflection sensitivity when pressing the cantilever onto a glass coverslip, which allowed the cantilever spring constant to be determined using the thermal noise method. For each MeHA gel, indentation force curves at six different locations on the gels were acquired. Before and during indentation experiments, gels were kept in PBS at 37 °C. To obtain the stiffness values from force curves, we used the PUNIAS software (http://punias.free.fr). Specifically, we corrected for baseline tilt, and used the linear fitting option for the Hertz model with a Poisson ratio of 0.5 on the indentation curve.

### Permeability of MeHA hydrogel cover

MeHA microwells covered with MeHA hydrogel were placed under a SP8 confocal microscope, and then immersed in cell culture medium containing 0.1 mg mL^−1^ GFP for 10 min. Images were taken at various time points and photon counting mode was used for quantitative analysis of the fluorescence intensity.

### Culturing of hMSCs and seeding into 3D microniche

hMSCs were obtained from Lonza and cultured in DMEM low glucose (Gibco) supplemented with 10% FBS (Gibco), 1% L-glutamine, and 1% Pen/Strep (Thermo fisher scientific). Cells were passaged before confluency and used at passage 6. Cells were seeded on the MeHA–Fn substrate containing microwells at a certain density in DMEM high glucose (GE healthcare) and incubated for 10–15 min; subsequently, the MeHA hydrogel was washed several times with a gentle flow of medium to remove non-adherent cells, and placed on top of a thin MeHA–Fn hydrogel lid (~ 30 µm height).

### Differentiation assays

Differentiation medium was composed of proliferation medium and osteogenic and adipogenic chemical supplements (5 × 10^−7^ M dexamethasone, 5 mM β-glycerolphosphate, 0.1 mM ascorbic acid-2-phosphate, 250 μM 3-isobutyl-1-methylxanthine, 5 μg mL^−1^ insulin, and 5 × 10^−8^ M rosiglitazone maleate, all from Sigma). hMSCs were cultured for 7 or 10 days in differentiation medium for osteogenic and adipogenic differentiation, respectively. Subsequently, all cells in microniches with different sizes and geometries were fixed with 4% PFA, the lid was removed afterward and cells were penetrated with 0.2% Triton X-100 for 10 min. ALP staining was performed by Fast Blue assay (naphthol-AS-MSC phosphate and Fast Blue RR, Sigma) in Tris−HCl buffer (pH 8.9) and incubated at 37 °C for 1 h. Oil Red O staining was performed by incubating cells with 1.8 mg mL^−1^ Oil Red O (Sigma) for 30−60 min at room temperature and then rinsing with 60% isopropanol (Sigma). Images were acquired on a Zeiss inverted microscope (Photometrics, USA).

### Cell staining

hMSC cells that adhered within MeHA–Fn microniches were fixed with 4% paraformaldehyde (Sigma) for 10 min, and then the lid of the microniches was carefully removed, followed by washing three times with PBS and then permeabilized with 0.2% Triton X-100 (Sigma) in reverse osmotic H_2_O, washed two times with PBS, and incubated with 1% bovine serum albumin (BSA) in PBS for 1 h. Subsequently, the cells were stained with phalloidin tetramethyl-rhodamine B isothiocyanate (TRITC) (Millipore, R415, 1:1000 in 1% BSA) for 1 h to visualize F-actin, Alexa fluor488–DNaseI (Invitrogen, D12371, 1:500) to visualize G-actin, anti-vinculin (Abcam, AB18058, 1:500) for FAs, anti-myosin IIa (Sigma, 150M4764, 1:500) for cell contractility, YAP/TAZ (Cell signaling, D24E4, 1:500), HDAC3 (Abcam, ab32369, 1:100) or active RhoA-GTP monoclonal antibody (NewEast Biosciences, 26904, 1:100), washed three times with PBS and stained with 4,6-diamidino-2-phenylindole (DAPI) (Millipore, 28718-90-3, 1:1000 in 1% BSA), Alexa-488 labeled goat anti-mouse (Thermo Fisher Scientific, A11029, 1:200) or anti-rabbit IgG (Thermo Fisher Scientific, A10040 or A21071, 1:200) for 1 h at room temperature, followed by three times PBS wash and one H_2_O wash. Images were taken within 24 h after staining using a Leica SP8 confocal laser scanning microscope (Leica, Germany). Myosin IIa motor activity was inhibited by treating cells with Bleb (Sigma) for 40 min. C3-exoenzyme (Cytoskeleton, Inc.) was used as an inhibitor of Rho GTPase. For quantitative imaging (F-actin, G-actin, and myosin IIa), images were taken with photon counting mode to make sure the intensity of fluorescent settings for all the images was the same.

### Cell viability analysis

To analyze cellular viability, a Live/Dead assay was performed with calcein AM and ethidium homodimer (Molecular Probes, Invitrogen detection technologies). These components were added to PBS at a concentration of 2 μg mL^−1^ and 4 μg mL^−1^, respectively. After removing the lid, the hydrogels containing cells were incubated in this solution for 30 min at room temperature and visualized under a Leica SP8 confocal microscope. Live cells stain green while dead cells take up the red dye.

### Cell proliferation assay

Cell proliferation was determined by EdU labeling. hMSCs were seeded in 3D microniches for 2 and 9 days, followed by treatment with 1 × EdU solution. When the incubation was up to 3 and 10 days, cells were fixed and permeabilized with 4% PFA and 0.1% Triton X-100, respectively. Following these processes, samples were treated according to the manufacturer’s protocol of Click-iT EdU Alexa Fluor-488 HCS Assay (Thermo Fisher Scientific). All images were collected by a Leica SP8 confocal microscope (Leica, Germany) with filters for DAPI and Alexa Fluor-488. For quantification, lower magnification (10× objective) fields were collected within regions of interest.

### RNA fluorescence in situ hybridization and imaging

To examine the mRNA expression in cells with different geometries and sizes, single-molecule mRNA FISH was performed on different samples. hMSCs in 3D microniches with different geometries and sizes were fixed with 4% paraformaldehyde (sigma) for 10 min; after removing the lid, samples were permeabilized with 70% ethanol before in situ hybridization. Afterward, samples were stained with oligonucleotide probes for RhoA labeled with Quasar 570 Dye, Arp2/3 mRNA with Atto 647, and TEAD mRNA with Quasar 570 Dye (Stellaris oligonucleotides, Biosearch Technologies). Oligonucleotide probe sequences used to assay RhoA, Arp2/3, and TEAD RNA abundance are RhoA: 5′-CCTGAAGAAGGCAGAGATATGGCAAACAGGATTGGCGCTTTTGGGTACAT-3′, Arp2/3: 5′-CAGCCAGCGCCCGCGATGAC-3′, and TEAD: 5-CTAGCTAGCAACATGGAAAGGATGAGCGACT-3. The poly(dT) probe that detects total mRNA polyA tails was purchased from GeneDetect. Subsequently, samples were washed with 2 × saline sodium citrate buffer (SSC) with 10% formamide (Ambion), and then 2 × SSC supplemented with DAPI to stain the cell nuclei. Cells in microniches were mounted in 2 × SSC and compressed between two cover slips for imaging. Single mRNA molecules were imaged using a 63× HC PL APO CS2, Na 1.40 objective on the DMi8 microscope of the Leica SP8 automated widefield fluorescence microscope equipped with a cooled DFC 420C, a cooled DFXC365 FX camera, and filter sets specific for each fluorophore. Images were taken as a series of optical z-sections (1 micron per section) spanning the vertical extent of each cell.

### Microscopy data analysis

All confocal images were taken with different z-stacks and overlaid in Fiji software with Image 5D plugin. The distance between two z-stacks was the same (1 µm) for all the samples. For quantitative analysis of the fluorescence intensity, images were taken by confocal microscope with photon counting mode to make sure all camera setting was identical. For generating heat maps of FAs staining, raw fluorescent images were aligned in Fiji and incorporated into a z-stack; the total average intensity per pixel of each cell in microwells was measured afterward to generate fluorescent heat map. For quantification of cell volume, project area (*A*_project_) of single cells was calculated by averaging the F-actin area of different z-stacks, the height (*H*) of cells was quantified from cross-section view of >30 cells, and the volume was then calculated as: *A*_project_ × *H*. Nuclear volume and spatial chromatin organization were measured by FIJI as described previously^11, 25^. For quantification of copy number from RNA FISH images, on collecting images of mRNA FISH samples, total mRNA spots were counted from different z-stacks using custom plugin in Fiji software. The mRNA concentration was calculated by diving the total mRNA spots into cell volume (RhoA, Arp2/3, polyA tails) or nuclear volume (TEAD), depending on the localization of mRNA.

### Statistics

Statistical analysis was performed with Origin software, and one-way analysis of variance (ANOVA) using a Tukey post-test for more than two variables was carried out. “Significant” and “very significant” differences were indicated by *(*P* < 0.05) or **(*P* < 0.01), respectively. All results were expressed as mean ± standard error.

### Data availability

The authors declare that the data supporting the findings of this study are available within the paper and its Supplementary Information files. Additional data are available from the corresponding author upon reasonable request.

## Electronic supplementary material

Supplementary Information
