# Supplementary material for: 3D microniches reveal the importance of cell size and shape
Source: Nat Commun. 2017 Dec 6;8:1962. doi: 10.1038/s41467-017-02163-2 (PMC5719012; doi:10.1038/s41467-017-02163-2)
Supplement: Supplementary file 1 — Supplementary Information [file 41467_2017_2163_MOESM1_ESM.pdf]

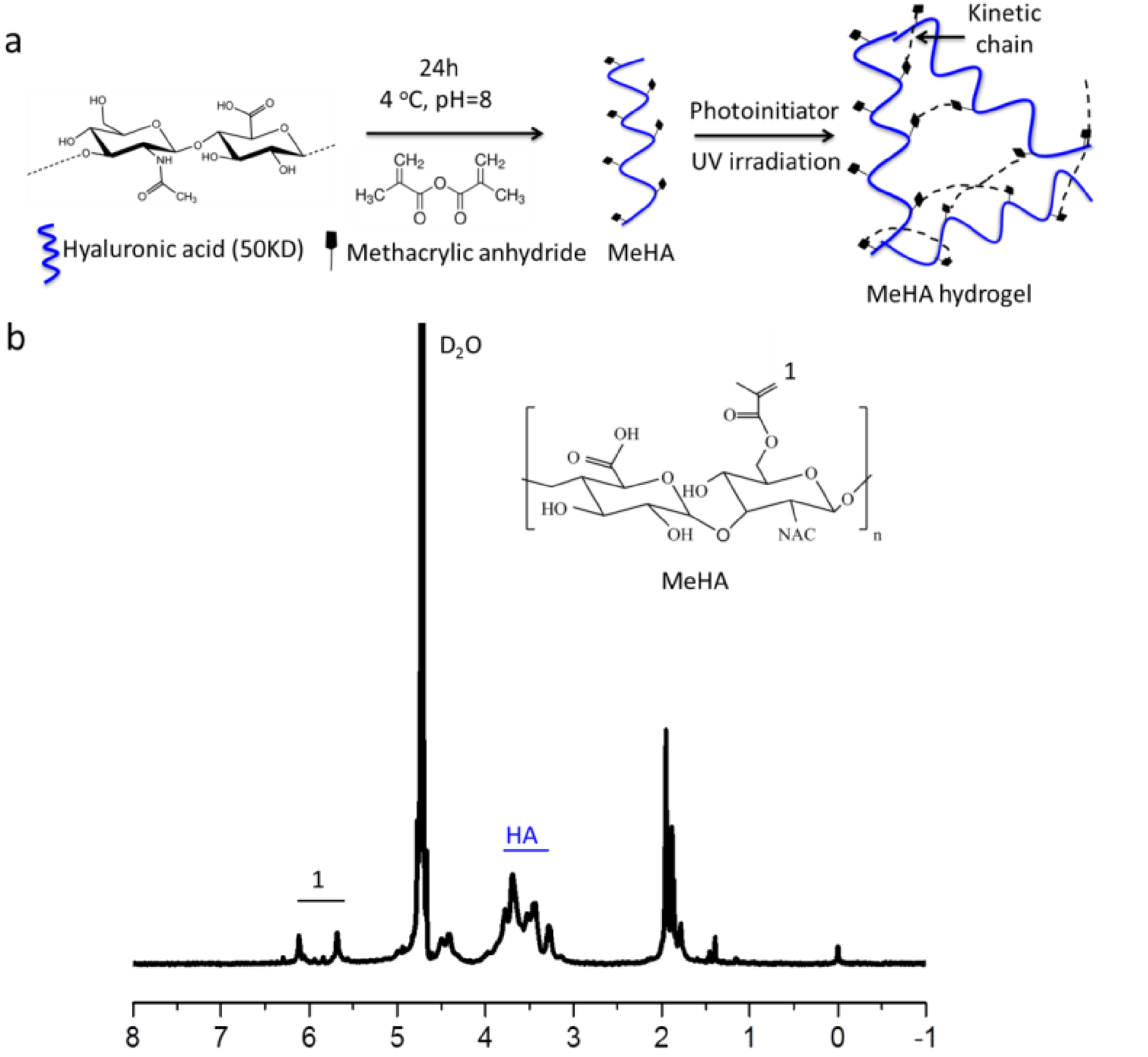

Supplementary Figure 1. a) Schematic of preparing MeHA and photopolymerization of MeHA. b) ^{1}H NMR spectrum is used to confirm MeHA. The degree of methacrylation was ~15%.

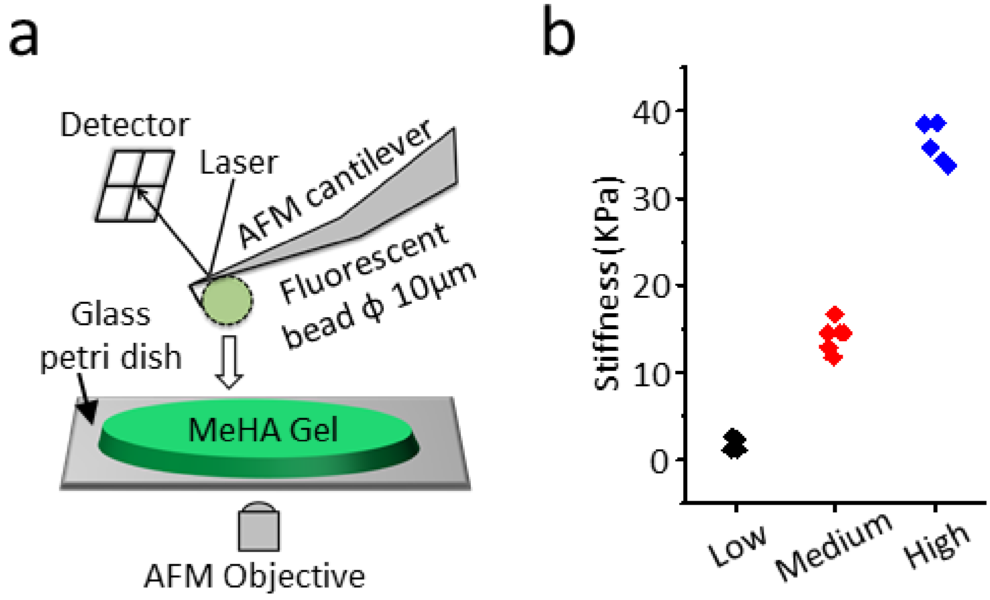

Supplementary Figure 2. a) Experimental setup of AFM-indentation based stiffness measurement for MeHA hydrogel. b) MeHA Hydrogel stiffness with varying macromer concentration (ranging from 2 - 10 wt%). The stiffness of MeHA gels in this study is ~10 kPa.

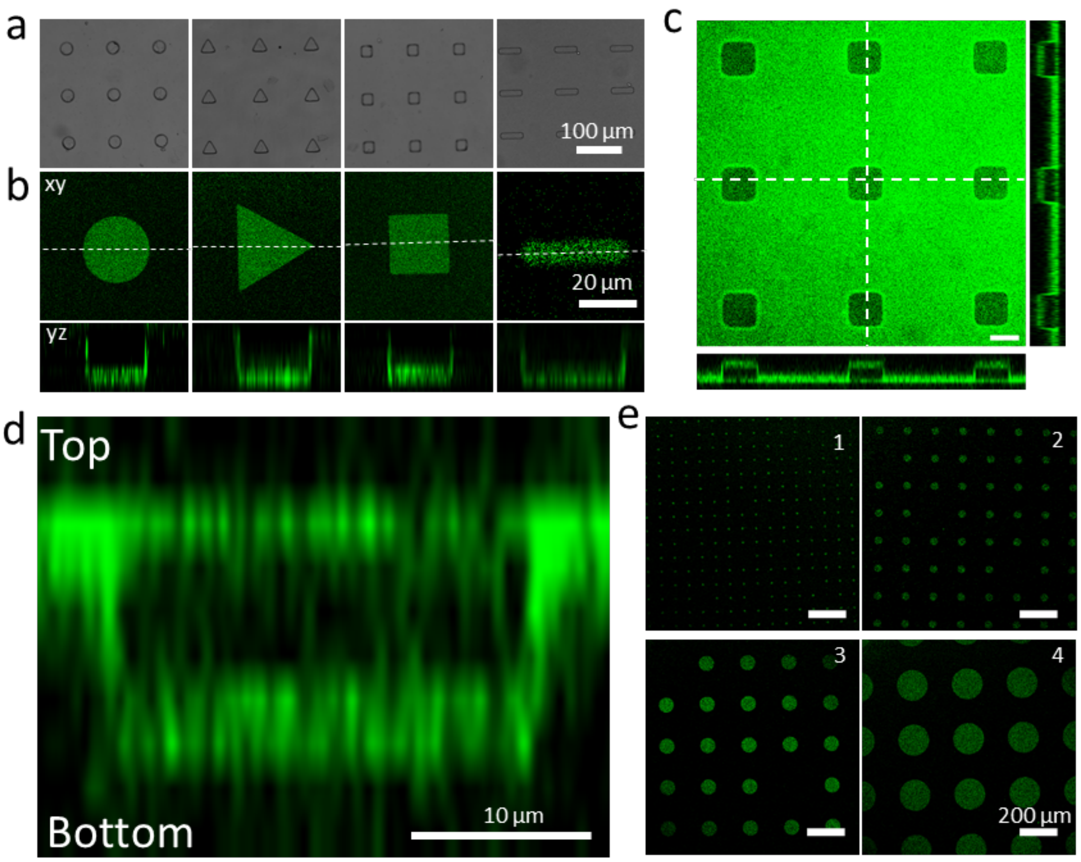

Supplementary Figure 3. a) Phase contrast images of MeHA hydrogel containing microwells of various geometries. b) Maximum intensity projections with different z-stacks and cross-sections of confocal images of microwells coated with Fn. c) Confocal image shows Fn distribution in microwells and on the surface of lid. Scale bar 20 µm. d) Confocal image (at larger magnification) shows homogeneous Fn distribution within 3D microniches from top to bottom. e) Confocal images of different sized microwells, the diameter for size 1: 22.6 µm, size 2: 45 µm, size 3: 70 µm, size 4: 125 µm.

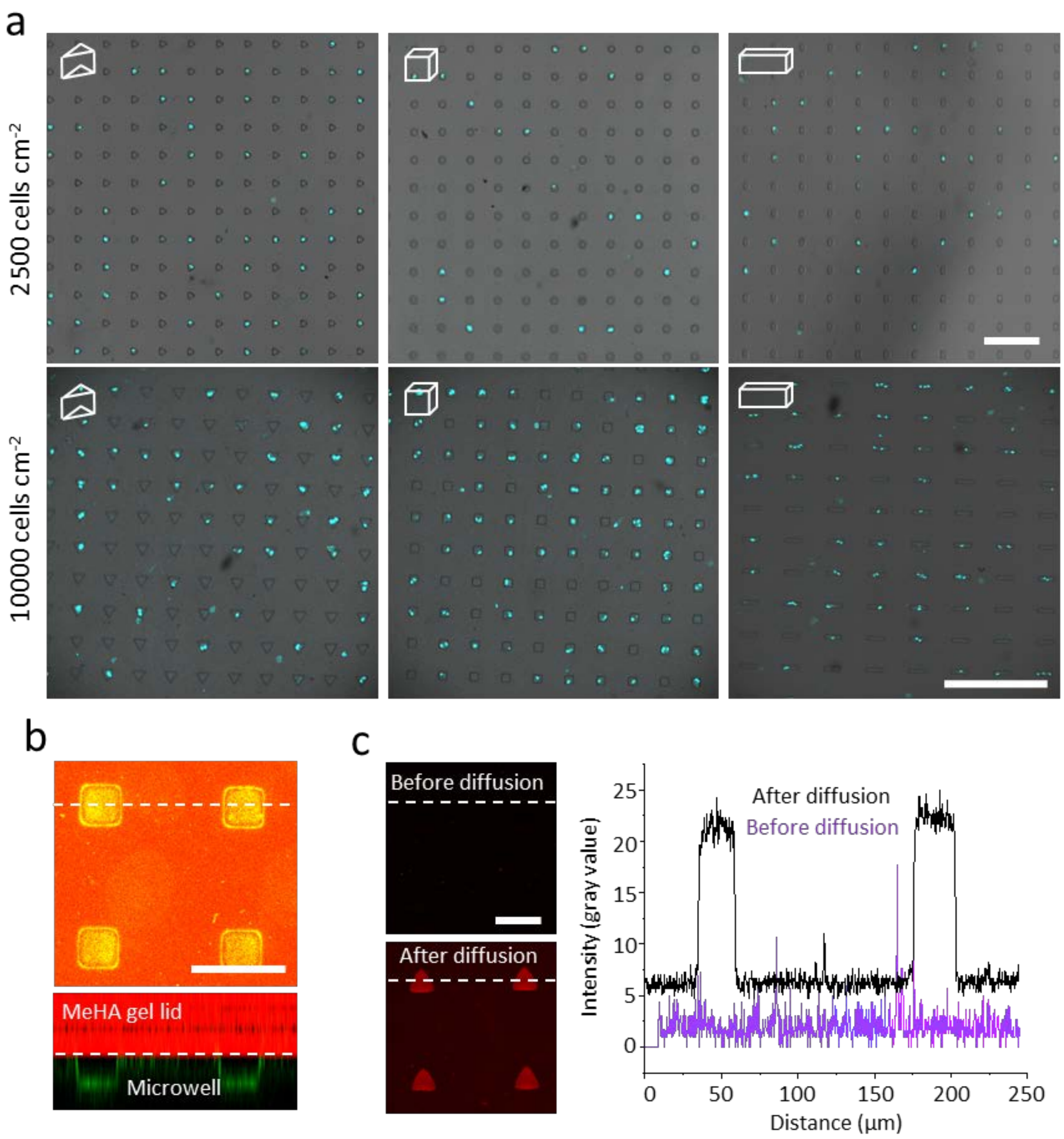

Supplementary Figure 4. a) Fluorescence image shows nuclear staining of single cells encapsulated in 3D microniche with different geometries (prism, cubic and cuboid) at different cell densities (2500 and 10000 cells cm^{-2}). scale bar 100 µm. b) Confocal image of MeHA hydrogel lid (red fluorescence) on MeHA hydrogel with microwells coated with Fn (green fluorescence), scare bar: 50 µm. c) Fluorescence intensity profiles (gray value) in microwells covered with MeHA hydrogel before and after immersed in GFP contained cell culture medium. Scale bar 50 µm.

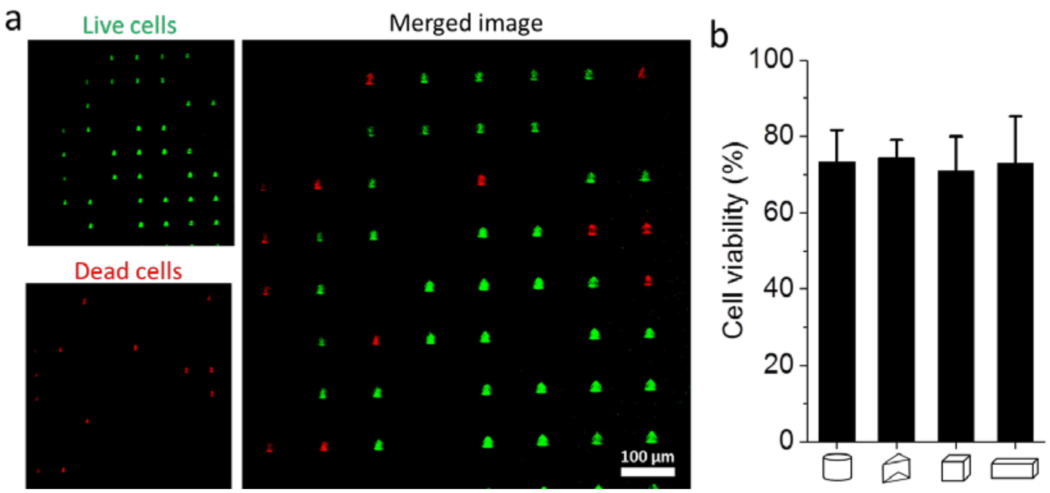

Supplementary Figure 5. a) Representative images of live/dead assay for hMSCs cultured in prismatic 3D microniche for 10 days. The cells were stained with calcein AM (green) and Ethidium homodimer-1 (red). b) Quantitative analysis of live/dead cell viability of cells cultured in microniches with different geometries after 10 days. mean ± s.d., n ≥ 4 regions of interest (ROI) with totals of 80-100 cells analyzed.

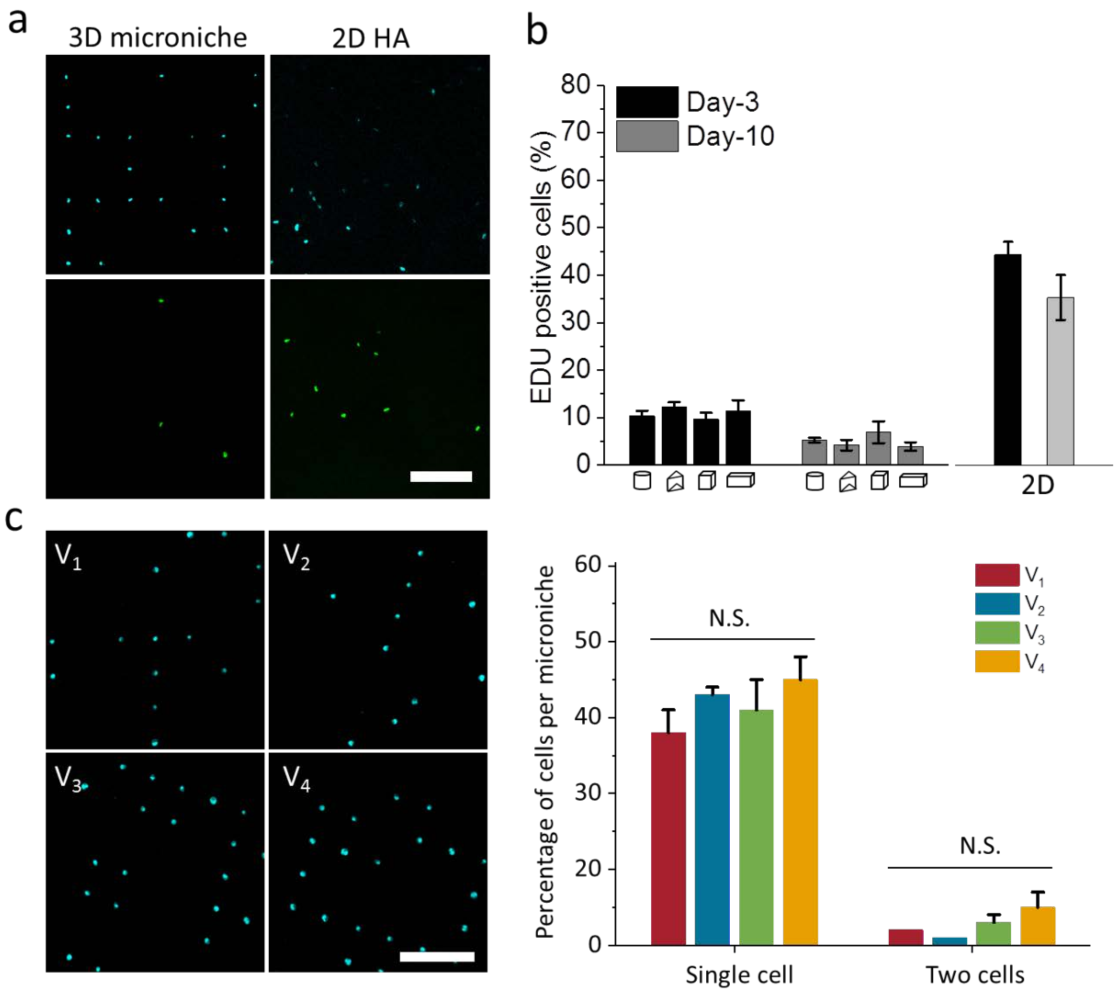

Supplementary Figure 6. a) Representative images of EdU assay in hMSCs cultured in 3D microniche and on 2D flat Fn coated MeHA hydrogel after 10 days. b) Proliferation of hMSCs over 3 and 10 days as determined by EdU incorporation. c) Percentage of one cell or two cells per cylindrical microniche with different volumes after 1 day culture. Scale bar 100 µm. mean ± s.d., $n \geq 5$ regions of interest (ROI) with totals of 100-150 cells analysed, $*P < 0.05$, N.S. means no significant differences.

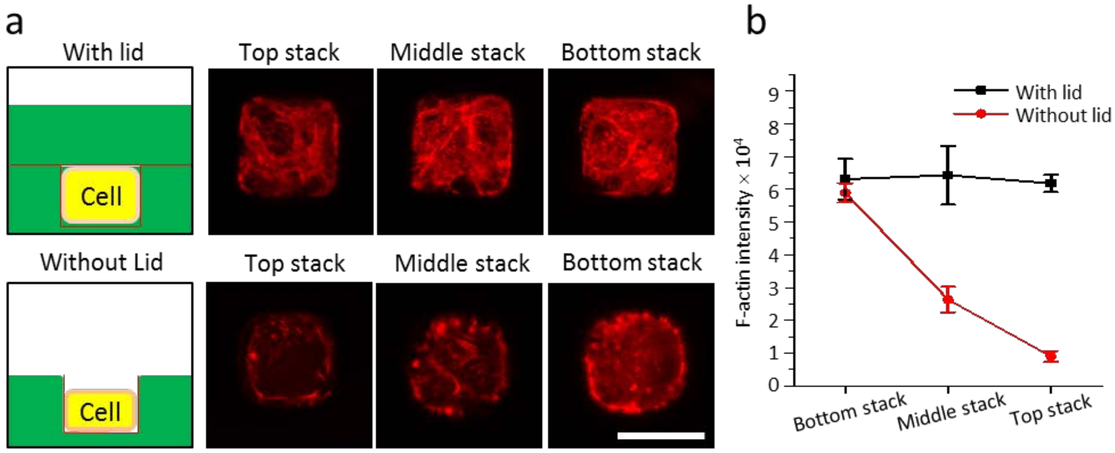

Supplementary Figure 7. a) F-actin staining for cells cultured in 3D microniche with and without lid on top. Scale bar 20 µm. b) To visualize the altered actin organization in microniche with and without lid on top, the fluorescent signals of actin (phalloidin 633) were quantified after 24 hours. mean ± s.d., n = 30-50 cells analysed.

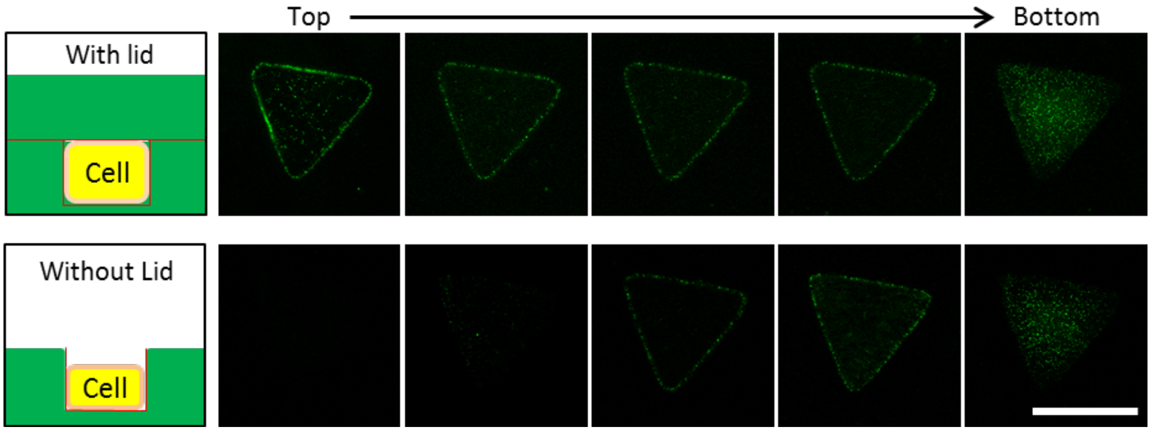

Supplementary Figure 8. β1 integrin staining for cells in microniche with and without lid on top. The height of microwells is 23 µm and the lateral dimension is 400 µm^2. Cells in 3D microniche with lid on top can form integrins from top to bottom. However, no integrin binding at the top surface was observed for cells in microwells without lid on top. The scale bar is 20 µm. The lid was not removed during integrin staining. Scale bar 20 µm.

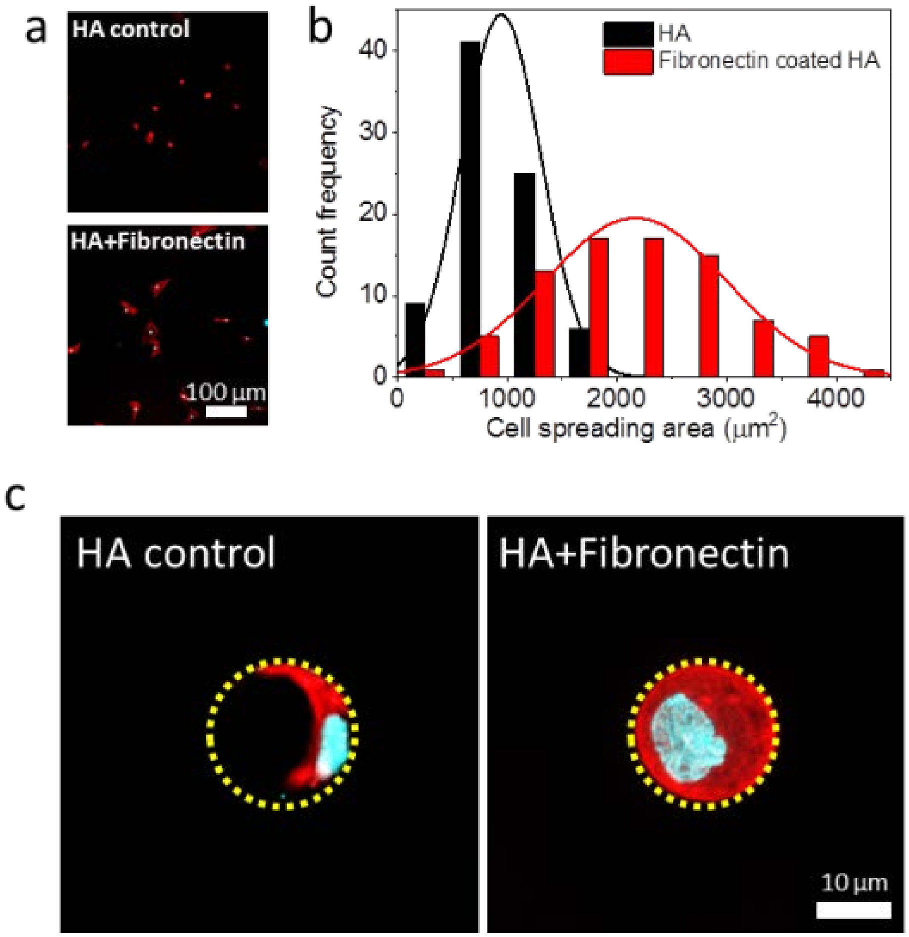

Supplementary Figure 9. a) Representative images of hMSCs on flat MeHA and fibronectin coated MeHA substrate. b) Normalized distribution of spreading area of hMSCs on MeHA substrate (n ≈150) and fibronectin coated MeHA substrate (n ≈ 170). c) Representative images of DAPI (blue) and F-actin (red) staining for single hMSCs cultured in 3D microniche for 12 hours. The project area and heigh of microniche is 400 μm^2 and 9 μm respectively.

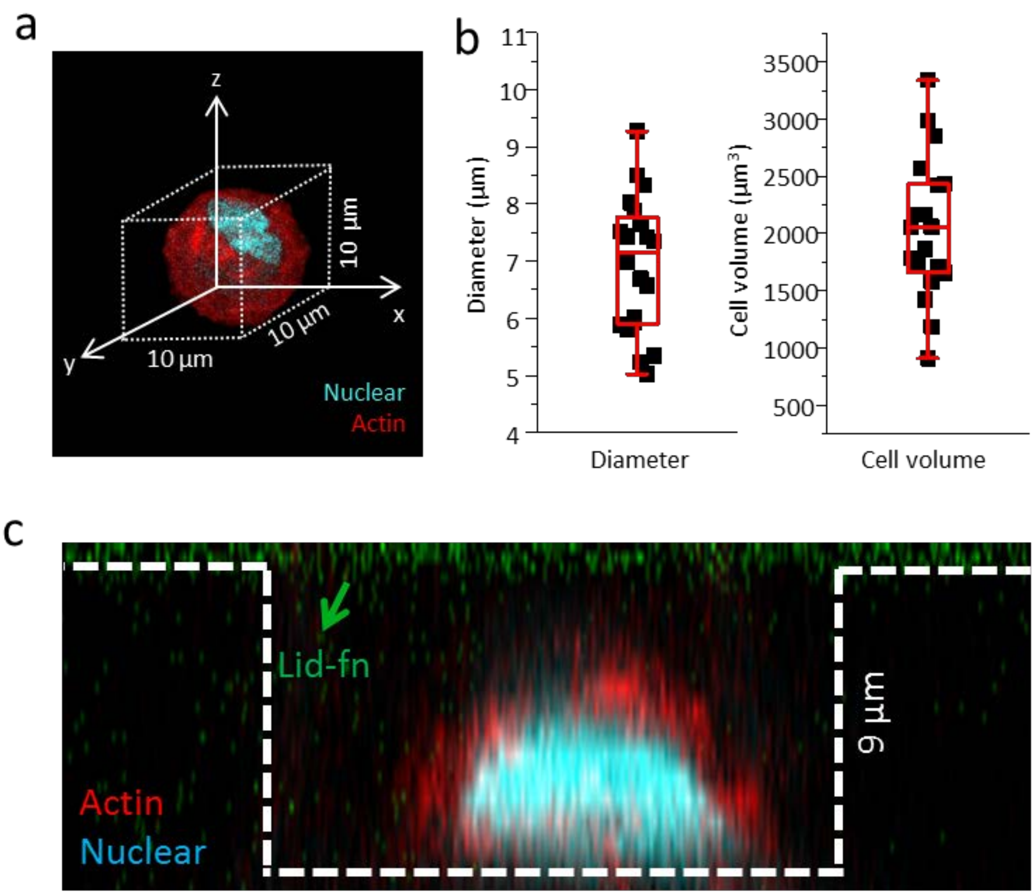

Supplementary Figure 10. a) A representative confocal image shows the dimension of a single hMSC cultured on MeHA hydrogel for 5 minutes, roughly the size of the cell in suspension. b) Quantification of diameter and cell volume for single hMSCs cultured on MeHA hydrogel for 5 minutes. c) A representative confocal image shows cross section view for cell in microwells with a lid on top. Cells were cultured in microwells for 15 minutes, then the microwell was sealed with a lid. Cells were stained with DAPI (blue) and phalloidin (red), lid was functionalized with FITC-fibronectin before putting on the microwells. The height and project area of this microniche is 9 µm and 400 µm^2. Dotted white line indicates the outline of microwells.

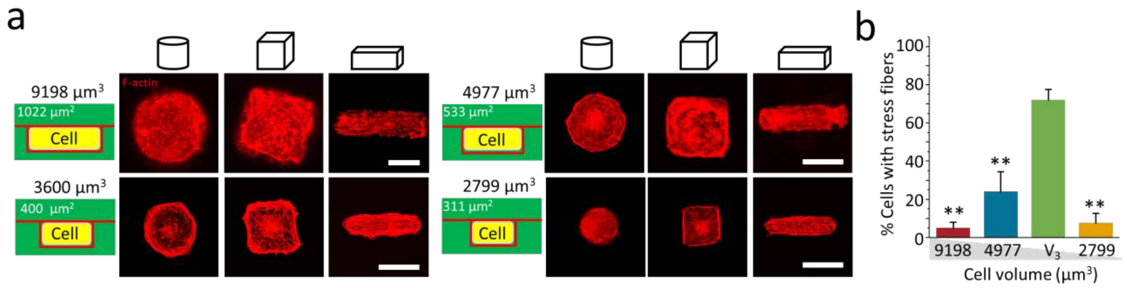

Supplementary Figure 11. a) F-actin (red) staining for cells seeded in microniches with different project area (1022, 533, 400, 311 μm^2) and different geometries (cylinder, cubic and cuboid) but the same height (9 μm). Scale bar 20 μm. b) Quantification of the number of cells forming stress fibres in 3D microniche with different volumes (different project areas but the same height). Data are shown as mean ± s.d. n=25-31 cells analysed for each data point and ∗∗P < 0.01.

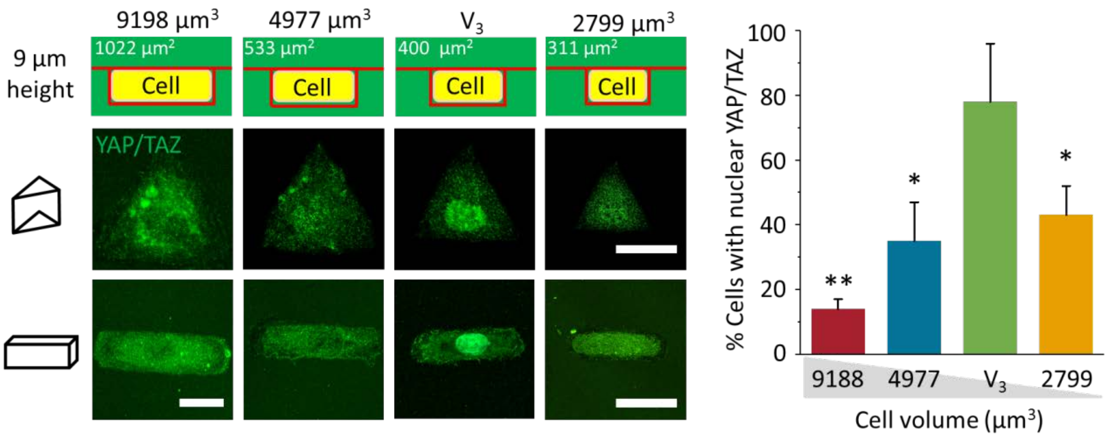

Supplementary Figure 12. Representative images and quantification of YAP/TAZ localization in hMSCs with different cell volumes (different project areas but the same height) after 24 h. Scale bar 20 μm. Data are shown as mean ± s.d. with totals of 20~30 cells analyzed, and *P < 0.05, **P < 0.01.

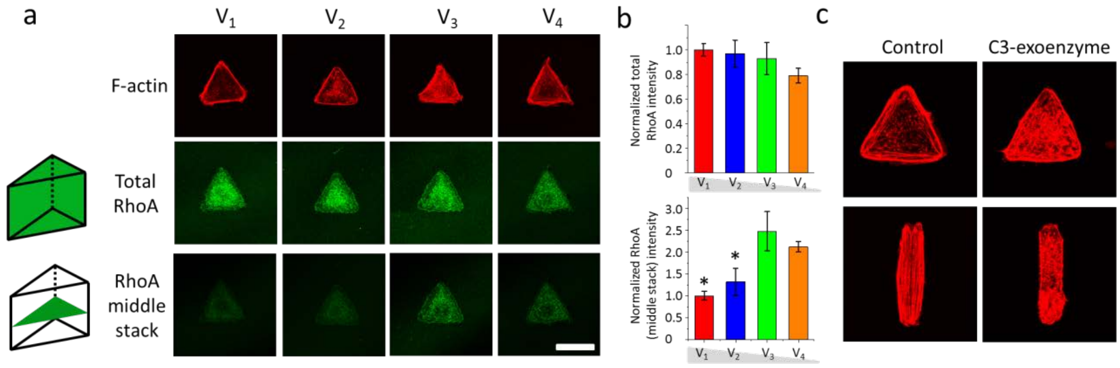

Supplementary Figure 13. a) Representative images of F-actin and RhoA staining for cells seeding in microniches with different volumes, total RhoA images were taken with different Z-stacks and overlaid in Fiji software with Image 5D plugin. The distance between two z-stacks was the same (1 µm) for all the sample. The middle stack for different cells was selected to compare RhoA intensity per stack. Scale bar: 20 µm. b) Quantification of total RhoA and middle stack RhoA intensity, normalized to V_1 cells. Data are shown as mean ± s.d. with totals of 15-26 cells analyzed, and *P < 0.05. c) Representative images of F-actin staining for V_3 cells treated with 10 µM C3-exoenzyme.

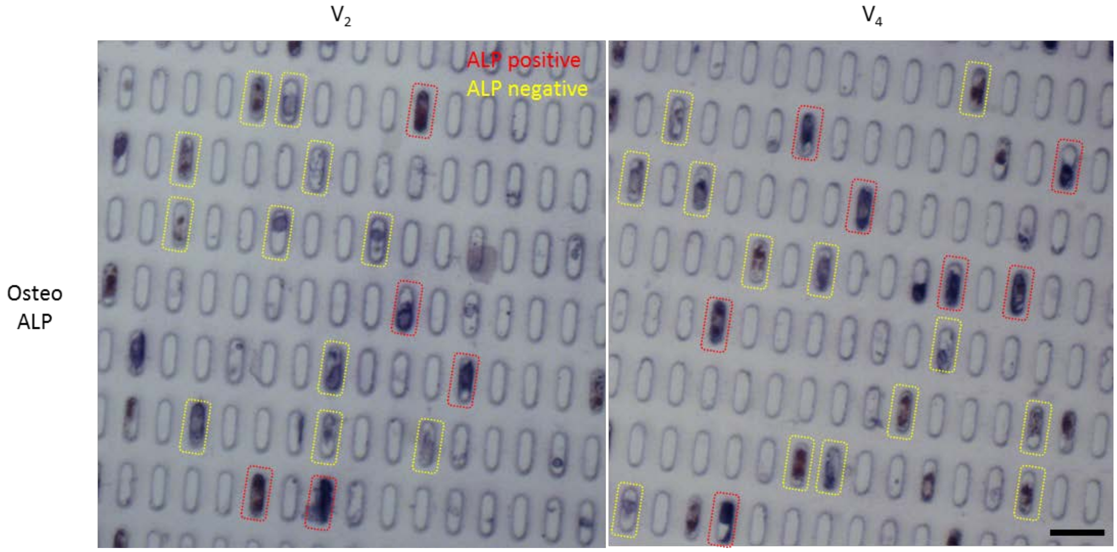

Supplementary Figure 14. Alkaline Phosphatase (ALP) staining for cells with different V_2 and V_4 volume. The ALP positive cells were determined by applying an optimal threshold to the image, ALP intensity above the threshold were determined as ALP positive. Scale bar 50 µm.
